# Comparison of Two Analytical Approaches to Dyadic Illness Management Among Patient–Caregiver Dyads in Type 2 Diabetes

**DOI:** 10.1097/NNR.0000000000000891

**Published:** 2026-02-06

**Authors:** Diletta Fabrizi, Michela Luciani, Maria Grazia Valsecchi, Davide Ausili, Paola Rebora

**Affiliations:** Diletta Fabrizi, PhD, RN, Postdoctoral Research Fellow, Michela Luciani, PhD, RN, Assistant Professor, Davide Ausili, PhD, RN, Professor, School of Medicine and Surgery, University of Milano-Bicocca, Monza, Italy; Maria Grazia Valsecchi, MS, Senior Professor, Paola Rebora, PhD, Associate Professor, Bicocca Bioinformatics Biostatistics and Bioimaging B4 Center, School of Medicine and Surgery, University of Milano-Bicocca, Monza, Italy; and Biostatistics and Clinical Epidemiology, Fondazione IRCCS San Gerardo dei Tintori, Monza, Italy

**Keywords:** caregiver, chronic disease, dyadic data analysis, multilevel analysis, latent class analysis

## Abstract

**Background:**

Patient self-care and caregiver contribution to self-care in chronic illnesses should be considered together as a dyadic phenomenon called “dyadic illness management.” The possibility of classifying dyadic engagement in chronic conditions care may uncover behavioral patterns useful for improving disease management. Mixed-effects models have been used to obtain dyadic scores, which serve as inputs for latent class analysis. However, the advantages of this approach over simpler synthetic dyadic measures remain unclear.

**Objective:**

The aim of this study was to compare two methods for obtaining dyadic scores to serve as inputs for identifying distinct patterns of dyadic illness management for type 2 diabetes (T2D) through latent class analysis.

**Methods:**

This work uses data from a cross-sectional study on 251 patients with T2D and their informal caregivers. Patient self-care and caregiver contribution to self-care were measured by the Self-Care of Diabetes Inventory and the Caregiver Contribution to Self-Care of Diabetes Inventory, respectively. To assess dyadic illness management, we first adopted the incongruence model, a mixed-effects model with a specific codification that enables the estimation of both the average and incongruence in the outcome within each dyad, through random intercepts and random slopes. These estimated coefficients were then used to perform a latent class analysis that was able to identify patterns of dyadic management. As an alternative approach, we calculated the dyadic average and incongruence using the raw means and the difference between patient and caregiver scores. These values were then used as inputs for latent class analysis.

**Results:**

The latent class analysis clustered the same dyads into the same classes across both approaches, with identical fit indices. The three-class model showed the best performance in terms of both fit and characterization of the dyads.

**Discussion:**

Mixed-effects models account for interdependence within the dyad and measurement error, returning predicted measures that are shrunk toward the overall mean. However, this approach yielded the same clusters as the simpler method based on observed measures.

The need to consider both the patient and the caregiver in the context of chronic illnesses has been highlighted by both theoretical and empirical evidence, sparking a growing interest in understanding how they manage illness together (Lyons & Lee, 2018). Indeed, informal caregivers, who provide unpaid assistance, have been shown to support patients with chronic conditions in adhering to complex self-care behaviors (Vellone et al., 2019). Hence, patient self-care and caregiver contribution to self-care in chronic illnesses should be considered a dyadic phenomenon, referred to as dyadic illness management, in which both members of the patient–caregiver dyad mutually influence each other (Lyons & Lee, 2018).

The identification of distinct and unobserved patterns of dyadic illness management could be highly valuable for studying chronic illness care, and several studies have performed it adopting the latent class analysis (Bonds et al., 2021; De Maria et al., 2023; Lee et al., 2015; Fabrizi et al., 2025; Lee & Lyons, 2019). The general recommendation in dyadic research, particularly in caregiving research, is to use a two-stage approach. This involves applying a mixed-effects model—the so-called incongruence model—to each dyad to estimate the dyadic outcome (e.g., dyadic engagement in type 2 diabetes care; Kenny et al., 2006; Lee et al., 2020; Lyons & Lee, 2020b; Sayer & Klute, 2005) and then using the estimated parameters as inputs for the latent class analysis (Bonds et al., 2021; De Maria et al., 2023; Lee et al., 2015; Lee & Lyons, 2019). Specifically, the incongruence model is a mixed-effects model that accounts for measurement error and the interdependence of data at the item level (Lee et al., 2015; Lyons et al., 2002). This model presents a codification that allows the estimation of the average level of the outcome (e.g., dyadic illness management) within the dyad (dyadic mean) from the intercept, and the gap between the two members of the dyad (dyadic incongruence) from the slope (Lyons & Lee, 2020b). The point estimates of the intercept and slope are then used as inputs in the latent class analysis.

However, the advantages of this two-stage approach over the simpler use of synthetic dyadic measures as inputs for latent class analysis (i.e., one-stage approach) remain unclear. Sayer and Klute (2005), in reviewing traditional methods for constructing dyadic measures from individual data, argued that approaches based on mean and difference scores have notable limitations. Specifically, mean scores are informative only when there is concordance between the two members’ responses, whereas difference scores, although they capture discrepancies, fail to account for the dyad’s location along the outcome scale. Nonetheless, these limitations appear to arise primarily when the two parameters are examined independently. In contrast, when mean and difference scores are considered jointly, the resulting information may not be deficient compared to that obtained through mixed models.

Given the complexity of the two-stage approach, involving mixed-effects models, which may hinder both the proper application of the method and the deep interpretation of the results, a comparison between the two approaches for obtaining dyadic measures to identify patterns of dyadic behavior is warranted. Such a comparison could delve into the strengths and limitations of each approach and provide guidance for future research. The aim of this study was to compare the two-stage approach with the direct use of mean and difference scores in latent class analysis for identifying distinct patterns of dyadic engagement in managing a chronic condition.

## METHODS

We used data from a multicenter cross-sectional study (Fabrizi et al., 2025) involving patients with type 2 diabetes (T2D) and their informal caregivers from four outpatient diabetes clinics in Northern Italy. The study received approval from the institutional review board of each participating center (Azienda Socio-Sanitaria Territoriale Grande Ospedale Metropolitano Niguarda, Milan, Italy; Azienda Socio-Sanitaria Territoriale Monza, Monza, Italy; Azienda Socio-Sanitaria Territoriale Garda, Brescia, Italy; Azienda Socio-Sanitaria Territoriale Lariana, Como, Italy), confirming that no further ethical approval was required. Further details were described elsewhere (Fabrizi et al., 2025).

### Sample

A sample of adult patient–caregiver dyads was recruited during outpatient visits. Patients were excluded if they had difficulties reading the study questionnaire, had been diagnosed with T2D for less than 1 year, were attending their first visit to the diabetes center, or had confirmed cognitive impairment. Caregivers were excluded if they had difficulties reading the study questionnaire or had confirmed cognitive impairment.

### Measurement

Patient self-care and caregiver contribution to self-care were measured using the Self-Care of Diabetes Inventory (SCODI; Ausili et al., 2017) and the Caregiver Contribution to Self-Care of Diabetes Inventory (CC–SCODI; Fabrizi et al., 2025), respectively, whose validity has been previously supported (Ausili et al., 2017; De Maria et al., 2022; Fabrizi et al., 2025). These two instruments consist of the same three scales (self-care/contribution to self-care maintenance, self-care/contribution to self-care monitoring, and self-care/contribution to self-care management, respectively), each assessing the same behaviors and differing only in the wording to reflect the patient or caregiver perspective. The self-care/contribution to self-care maintenance scale assesses behaviors aimed at maintaining physical and emotional stability (e.g., adherence to diet, medications, and physical activity recommendations). The self-care/contribution to self-care monitoring scale evaluates behaviors aimed at recognizing bodily signs and symptoms (e.g., monitoring blood glucose, body weight, feet, and symptoms of hypoglycemia or hyperglycemia). The self-care/contribution to self-care management scale appraises behaviors taken in response to signs and symptoms (e.g., managing episodes of hypoglycemia and hyperglycemia; Ausili et al., 2017; Riegel et al., 2012). Each item is rated on a 5-point Likert-type scale from “*never*” to “*always*,” and each scale yields a standardized score ranging from 0 to 100, where higher scores indicate higher levels of self-care or contribution to self-care (Ausili et al., 2017; Fabrizi et al., 2025).

### Statistical Analysis

First, we adopted the two-stage approach, in which dyadic illness management was estimated as random intercepts and slopes in the incongruence model (Lyons & Lee, 2020b; Raudenbush & Bryk, 2002; Sayer & Klute, 2005). We fitted three incongruence models, one for each pair of SCODI/CC–SCODI scales, as follows:

(1) $${\mathrm{Dyadic illness management}}_{\mathrm{ij}}=\beta_{\mathrm{0j}}+\beta_{\mathrm{1j}}I_{\mathrm{ij}}+r_{\mathrm{ij}}$$

where *dyadic illness management*_*ij*_ represents the self-care/contribution to self-care score for person *i* in dyad *j*, and *I*_*ij*_ is an indicator variable indicating which dyad member reported the score (codified as −0.5 for the caregiver and 0.5 for the patient; Lyons & Lee, 2020b; Raudenbush & Bryk, 2002). Under this codification, the intercept (*β*_*0j*_) represents the expected mean score of dyadic engagement in the dyad *j*, and the slope (*β*_*1j*_) the expected incongruence in dyadic engagement, reflecting the magnitude and direction of the discrepancy in the outcome between the two members of the dyad (Lyons & Lee, 2020b). The coefficients *β*_*0j*_ and *β*_*1j*_ are conceived as varying randomly across dyads (i.e., random effects reflecting the variability around the average level of dyadic illness management mean score and incongruence) and are assumed to follow a normal distribution with a mean of zero and variance σ^2^ (Raudenbush & Bryk, 2002; Sayer & Klute, 2005). The residuals (*r*_*ij*_) capture measurement error and are also assumed to follow a normal distribution with a mean of zero and variance σ^2^ (Raudenbush & Bryk, 2002; Sayer & Klute, 2005). The incongruence model corresponds to the first step—within dyad—and provides two latent variables for each SCODI/CC–SCODI scale. These latent variables characterize each dyad by the intercept, representing the dyadic average level of the outcome, and the slope, representing the level of incongruence in the outcome between the two members of the dyad (Lyons & Lee, 2020a, 2020b; Sayer & Klute, 2005).

To identify patterns of dyadic engagement in T2D care, we then performed a latent class analysis (De Maria et al., 2023; Lee et al., 2015, 2020; Fabrizi et al., 2025). For each dyad, we included as inputs the previously predicted dyadic averages and incongruence for each SCODI/CC–SCODI scale (i.e., two predicted random effects for each of the three scales, resulting in six parameters total). The mean dyadic averages and incongruences for each identified class were estimated descriptively. Therefore, no confidence intervals were reported, as the latent classes were derived from an exploratory clustering procedure and may not reflect true population features.

As an alternative approach, for each SCODI/CC–SCODI scale, we calculated the dyadic average and incongruence directly from the raw patient–caregiver means and differences between patient and caregiver scores. These six measures were then used as inputs for a second latent class analysis. We derived the mathematical relationship between dyad-level estimates (i.e., dyadic average and incongruence) obtained from the two approaches for each SCODI/CC–SCODI scale, displayed their values in dispersion plots, and computed the Pearson's correlation coefficient.

To guide model selection (i.e., the optimal number of classes) for the latent class analysis, we evaluated the following fit indices: Bayesian information criteria (the lower the better), minor posterior probabilities (close to 1.0 for the most likely class), class sizes (not smaller than 5% of the sample), entropy (close to 1.0), Lo–Mendell–Rubin adjusted likelihood ratio test, and the parametric bootstrap likelihood ratio test (Lee et al., 2020; Ram & Grimm, 2009).

## RESULTS

A total of 251 patient–caregiver dyads were enrolled. Patients were mostly male (55%) with a mean age of 72 years (*SD*=10). Caregivers were predominantly female (71%) with a mean age of 63 (*SD*=13). Patients’ mean scores in the SCODI scales were: 77 (*SD*=14) for self-care maintenance, 65 (*SD*=23) for self-care monitoring, and 51 (*SD*=30) for self-care management. Caregivers’ mean scores in the CC–SCODI scales were 56 (*SD*=26) for caregiver contribution to self-care maintenance, 52 (*SD*=29) for caregiver contribution to self-care monitoring, and 49 (*SD*=34) for caregiver contribution to self-care management. The dyadic mean scores were 66 (*SD*=23) for self-care maintenance behaviors, 58 (*SD*=27) for self-care monitoring behaviors, and 50 (*SD*=32) for self-care management behaviors.

**FIGURE 1:**
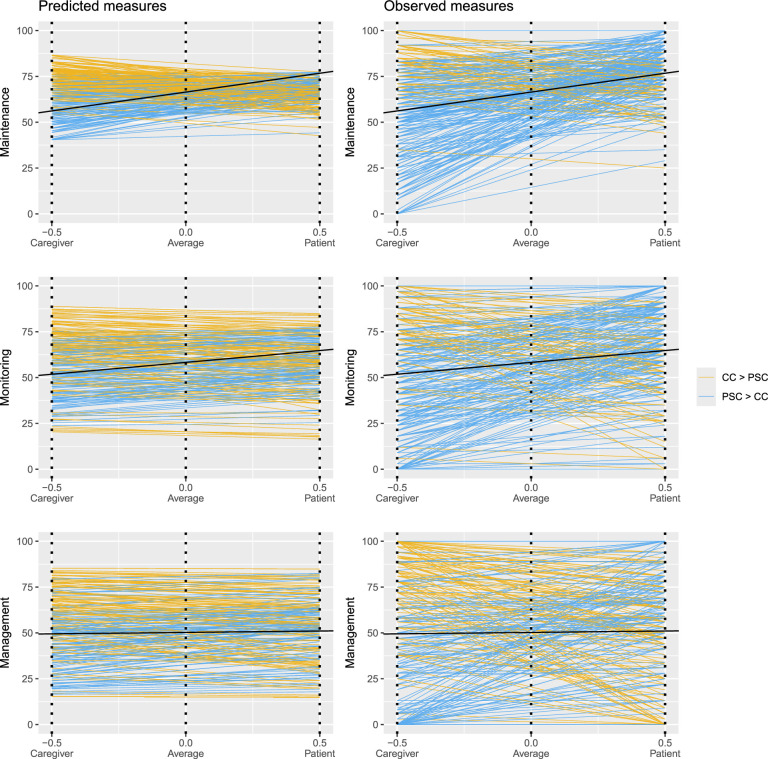
Predicted (two-stage approach) and observed (one-stage approach) dyadic engagement in self-care maintenance, monitoring, and management behaviors for each dyad (*n*=251). Each line coincides with one dyad. For each dyad, −0.5 on the *x*-axis coincides with the caregiver contribution to self-care maintenance, monitoring, or management score; 0.0 on the *x*-axis coincides with the mean dyadic engagement in self-care maintenance, monitoring, or management score. 0.5 on the *x*-axis coincides with the patient self-care maintenance, monitoring, or management score. Yellow lines denote that the caregiver contribution score is higher than the patient self-care score. Blue lines denote that the patient self-care score is higher than the caregiver contribution score. The black line coincides with the overall dyadic mean (fixed effects). *Note.* CC=caregiver contribution to patient self-care; PSC=patient self-care.

Figure 1 shows patient (*I*_*ij*_=0.5) and caregiver (*I*_*ij*_=-0.5) scores as predicted by the incongruence model (i.e., first step of the two-stage approach, left side) and as observed (i.e., raw mean and difference scores, right side) for each dyad. As expected, the dyadic averages and incongruences in type 2 diabetes management predicted by the models’ coefficients shrunk toward the overall mean (i.e., fixed effects), compared with the observed values.

Using the point estimates from incongruence models (two-stage approach) as input variables, the latent class analysis model with three classes was the one that, in addition to displaying the highest entropy and good performances in further fit indices (Table 1), was capable of clearly differentiated class profiles. Class 1 clustered 14% of dyads (*n*=34), class 2 clustered 61% of dyads (*n*=154), and class 3 clustered 25% of dyads (*n*=63).

**TABLE 1:** Performance Assessment of Latent Class Analysis Models With Increasing Number of Classes

	Performance Assessment Criteria					
No. classes	BIC	<Post. prob.	<Class size	Entropy	LMR-LRT (*p*)	PBLRT (*p*)
2	13778.782	.941	.35	.828	<.001	<.001
3	13726.389	.910	.14	.871	.0190	<.001
4	13692.062	.864	.14	.813	.0161	<.001
5	13658.800	.871	.10	.838	.0988	<.001

*Note.* Fit indices were identical when performing latent class analysis using either the coefficients from incongruence models or the observed measures as input. BIC=Bayesian Information Criteria (the lower the better); <Post. Prob=Minor posterior probability (the average of latent class probabilities for the most likely latent class membership by latent class. It must be close to 1); <Class size=minor class size (it must be not less than 5% of the sample); entropy=index of model Fconvergence (it must be close to 1); LMR-LRT (*p*)=*p* value of the Lo–Mendell–Rubin adjusted likelihood ratio test (if *p*>.05, the identified model it is not better compared with the previous one (number of classes - 1); PBLRT (*p*)=*p* value of the parametric bootstrapped likelihood ratio test (if *p*>.05, the identified model it is not better compared with the previous one (number of classes - 1).

**FIGURE 2:**
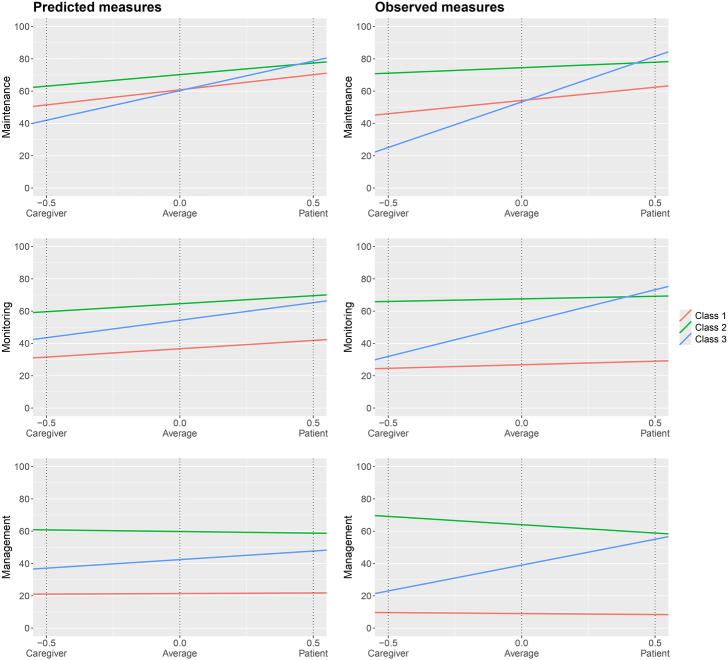
Comparison of dyadic engagement in self-care/contribution to self-care behaviors in the classes identified by latent class analysis, using as input variables either coefficients estimated by mixed-effects models (two-stage approach, left) or observed measures (one-stage approach, right). *Note*. −0.5 on the *x*-axis coincides with the estimated mean of caregiver contribution to self-care maintenance, monitoring, or management behaviors in each class; 0.0 on the *x*-axis coincides with the estimated mean dyadic engagement in self-care maintenance, monitoring, or management behaviors in each class. 0.5 on the x-axis coincides with the estimated mean of patient self-care maintenance, monitoring, or management behaviors in each class.

The left side of Figure 2 presents the results of the latent class analysis across the three self-care scales. Class 1 dyads were mostly characterized by low dyadic averages with small dyadic incongruences in illness care engagement. Class 2 dyads were mostly characterized by high dyadic averages with small dyadic incongruences. Class 3 dyads were mostly characterized by intermediate dyadic averages and large dyadic incongruences, with patients reporting higher scores than caregivers.

**FIGURE 3:**
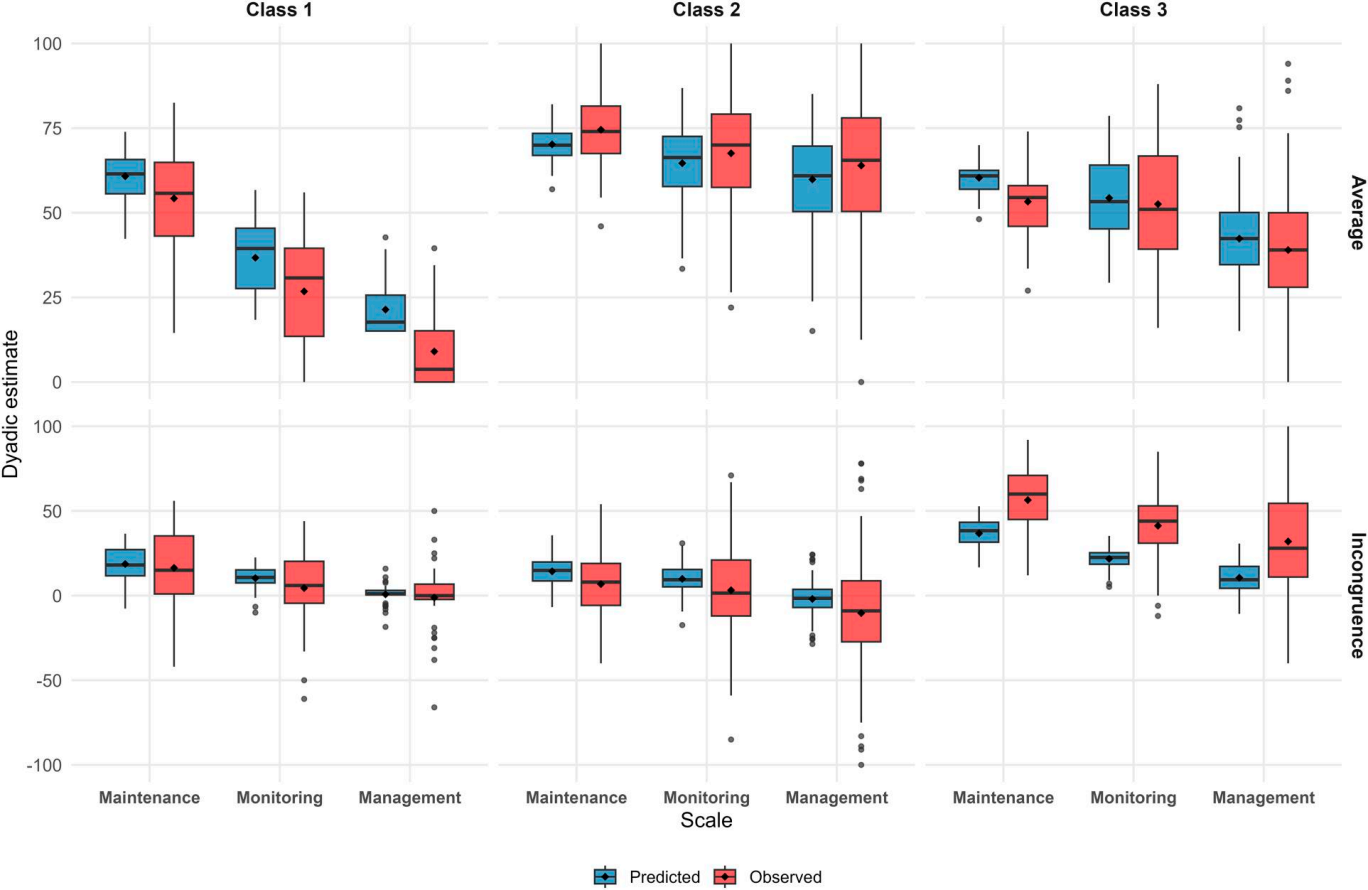
Dyadic average and incongruence distribution by predicted measures (two-stage approach) and observed measures (one-stage approach) in self-care maintenance, monitoring, and management scores for each identified class. *Note*. Boxplots show predicted (blue) and observed (red) dyadic estimates for self-care maintenance, monitoring, and management across the three identified classes. Upper panels display average scores, lower panels display incongruence scores. Black diamonds indicate mean values.

When the alternative (one-stage) approach was applied using the raw mean and difference between patient and caregiver engagement scores as inputs for the latent class analysis, the dyads were clustered identically to the two-stage approach, and the fit indices remained unchanged (Table 1). Indeed, the ordinal relationship between observed and predicted values for each dyad was preserved (Raudenbush & Bryk, 2002), indicating that the incongruence model had no effect on class membership. The only difference between the two approaches lies in the estimated self-care/contribution to self-care scores within each class (see comparison in Fig. 2 and Table 2), due to the shrinkage effect introduced by the incongruence models in the two-stage approach (Supplemental Digital Content [SDC] 1, http://links.lww.com/NRES/A595). Nevertheless, overall class trends were similar across both approaches. Furthermore, the correlation coefficient (*r*) between the observed dyadic means/differences and the corresponding mixed-model estimates was 1.00 for all scales (Supplemental Figure 1 in SDC 2, Supplemental Digital Content 2, http://links.lww.com/NRES/A596), indicating a perfect deterministic correspondence between the one-stage and two-stage estimates at the dyad level, as defined by the formula provided in SDC 1, Supplemental Digital Content 1, http://links.lww.com/NRES/A595. Table 2 and Figure 3 report the dyadic average and incongruence (from the predicted values in the two-stage approach) and the dyadic mean and difference (from the observed values in the one-stage approach) for self-care maintenance, monitoring, and management scores across the identified classes.

**TABLE 2:** Dyadic Average and Incongruence by Predicted Measures (Two-Stage Approach) and Dyadic Mean and Difference by Observed Measures (One-Stage Approach) in Self-Care maintenance, Monitoring, and Management Scores for Each Identified Class

		Class 1	Class 2	Class 3
	*n* (%)	34 (13.5)	154 (61.4)	63 (25.1)
		*M* (*SD*)		
Two-stage approach: predicted measures				
Maintenance	Dyadic average	60.8 (7.1)	70.2 (4.5)	60.3 (4.5)
	Dyadic incongruence	18.7 (10.7)	14.3 (8.7)	36.7 (9.0)
Monitoring	Dyadic average	36.7 (10.8)	64.6 (11.4)	54.4 (11.8)
	Dyadic incongruence	10.3 (6.9)	9.9 (8.2)	21.7 (6.1)
Management	Dyadic average	21.4 (7.8)	59.8 (13.8)	42.4 (13.5)
	Dyadic incongruence	0.7 (6.2)	−2.0 (9.5)	10.6 (9.1)
One-stage approach: observed measures				
Maintenance	Dyadic mean	54.2 (15.3)	74.5 (9.7)	53.3 (9.7)
	Dyadic difference	16.4 (23.6)	6.8 (19.2)	56.4 (19.9)
Monitoring	Dyadic mean	26.8 (15.8)	67.6 (16.7)	52.6 (17.3)
	Dyadic difference	4.4 (22.2)	3.2 (26.6)	41.3 (19.8)
Management	Dyadic mean	9.0 (11.1)	64.0 (19.8)	39.0 (19.3)
	Dyadic difference	−1.2 (20.8)	−10.3 (32.1)	32.0 (30.6)

*Note.* *SD*=standard deviation.

Positive values of dyadic incongruence/difference denote that the patient score is, on average, higher than the caregiver score. Negative values of dyadic incongruence/difference denote that the caregiver score is, on average, higher than the patient score.

## DISCUSSION

We aimed to compare two analytical methods for deriving dyadic scores as input variables to identify distinct patterns of dyadic illness management. When used to identify patterns of dyadic engagement, the dyadic averages and incongruences predicted by the incongruence (mixed-effects) models showed no advantage over directly using the observed means and differences. Indeed, dyads clustered in the same classes under both approaches. This finding is noteworthy, as it suggests that this type of dyadic data may be approached more simply when the primary aim is class identification.

Since the ordinality of scores within each dyad remained unchanged (Raudenbush & Bryk, 2002), the clustering was identical across the two approaches, as were the fit indices for models with varying numbers of classes. Indeed, as highlighted by the mathematical relationship and the correlation analysis, this deterministic correspondence was expected given the data structure: each dyad contributed exactly two observations (patient and caregiver), allowing the mixed-effects model to reproduce the observed mean and difference values with no residual variability. Under complete data and one observation per dyad member, the model-based dyadic estimates are essentially a linear transformation of the observed values. Importantly, in mixed-effects models, empirical Bayes shrinkage pulls dyad-specific estimates toward the population mean based on the ratio of between-dyad to within-dyad variances: when variability between dyads is high, the dyads differ considerably, so the estimates remain close to the observed values with minimal shrinkage. In contrast, when variability between dyads is low and the dyads are more similar, the estimates are pulled more strongly toward the overall mean, reflecting greater shrinkage (Bao & Booth, 2024).

In our case, the application of mixed-effects models led to an overall attenuation of between-dyad differences, resulting in the loss of a significant portion of the heterogeneity that we aimed to explain through the latent class analysis (Copas, 1983; Lee et al., 2020). This result is important to consider because classes identified using predicted values (two-stage approach) appeared more similar to each other compared with the clearer characterization obtained using observed values (one-stage approach). This raises a key question: Is it worthwhile to lose information if the result in terms of class structure remains unchanged? One might argue that applying mixed-effects models with covariates (i.e., patient, caregiver, or dyad characteristics) could yield different results. However, identifying patterns of dyadic engagement in illness care net of these characteristics (i.e., by including covariates in mixed-effects models) would result in the loss of critical information useful for characterizing patterns. Indeed, the result of latent class analysis performed using coefficients from a mixed-effects (incongruence) model with covariates would be the identification of classes not solely based on dyadic engagement in care, but also in relation to other characteristics, making a subsequent evaluation of potential differences between classes meaningless. While a more useful strategy would be to identify patterns of dyadic engagement first, and then examine whether patient, caregiver, or dyad characteristics differ across the identified classes (De Maria et al., 2023; Iovino et al., 2021) or whether specific attributes are associated with increased likelihood of class membership (Lee et al., 2015).

Notably, if the research objective shifts from pattern identification to examining associations between independent variables (at the patient, caregiver, or dyad level) and dyadic illness management outcomes, then the use of mixed-effects models becomes essential and undoubtedly more appropriate (Kenny et al., 2006; Raudenbush & Bryk, 2002; Sayer & Klute, 2005).

While both approaches yielded identical clustering results in our complete data set, another practical consideration concerns missing data. Although there were no missing values in the present study, mixed-effects models may offer an advantage in this regard, as they allow the estimation of dyad-level random effects using information from both the specific dyad and the overall sample distribution. This feature enables unbiased estimation under the missing-at-random assumption and represents a potential strength of the mixed-model approach.

### Limitations and strengths

This methodological comparison was conducted in a specific context (T2D) with a relatively limited sample size. Furthermore, the latent class analysis conducted here is exploratory in nature. As such, the extracted classes should not be interpreted as definitive population subgroups. The stability and replicability of the class solution were not formally tested, which would require resampling procedures such as bootstrapped latent class analysis. However, the study’s aim was to illustrate broader methodological considerations in a real-world setting. The main result—identical class membership across the two approaches—is theoretically supported and would likely hold in larger samples and other clinical contexts (Raudenbush & Bryk, 2002).

## CONCLUSION

We compared two analytical approaches to dyadic data analysis, which were then used to identify patterns of dyadic engagement in T2D. The first (two-stage) approach, which utilized mixed-effects models, accounted for within-dyad interdependence and measurement error, producing predicted values that were shrunk toward the overall mean. However, it yielded the same clusters as the simpler (one-stage) approach, which was based on the raw dyadic mean and difference, because the ordinal structure of scores remained unchanged between the predicted and observed values. These findings underscore the need for further exploration of dyadic data analysis, particularly given their potential for clinical application and for tailoring interventions based on patterns of dyadic engagement in illness care.

## Supplementary Material
