# Supplementary material for: Comparison of Two Analytical Approaches to Dyadic Illness Management Among Patient–Caregiver Dyads in Type 2 Diabetes
Source: Nurs Res. 2026 Feb 6;75(3):206–13. doi: 10.1097/NNR.0000000000000891 (PMC13098665; doi:10.1097/NNR.0000000000000891)
Supplement: Supplementary file 1 [file nnr-75-206-s001.pdf]

SUPPLEMENTAL DIGITAL CONTENT 1

Title

Comparison of Two Analytical Approaches to Dyadic Illness Management among Patient–Caregiver Dyads in Type 2 Diabetes

Relationship between observed and predicted dyadic average and incongruence

Model (1) can be specified as two levels:

Level 1 (within-dyad): $Y_{ij} = \beta_{0j} + \beta_{1j}I_{ij} + r_{ij}$, where $r_{ij} \sim N(0, \sigma^2)$
Level 2 (between-dyad): $\beta_{0j} = \gamma_{00} + u_{0j}$, where $u_{0j} \sim N(0, \tau_{00})$
$\beta_{1j} = \gamma_{10} + u_{1j}$, where $u_{1j} \sim N(0, \tau_{11})$

where Y_{ij} represents the self-care/contribution to self-care score for person *i* in the dyad *j* and I_{ij} is an indicator variable representing the dyad member reporting the score (codified as -0.5 for the caregiver and 0.5 for the patient) (Lyons & Lee, 2020a; Raudenbush & Bryk, 2002).

The BLUP (Empirical Bayes) estimates of predicted dyadic average and incongruence shrink the observed values toward the population means:

$$\hat{\beta}_{0j}^{\ BLUP} = \hat{\gamma}_0 + \lambda_0(\hat{\beta}_{0j}^{\ O} - \hat{\gamma}_0)$$
$$\hat{\beta}_{1j}^{\ BLUP} = \hat{\gamma}_1 + \lambda_1(\hat{\beta}_{1j}^{\ O} - \hat{\gamma}_1)$$

Where:

$\hat{\beta}_{0j}^{\ O} = (Y_{1j} + Y_{2j})/2$ (within-dyad mean)

$\hat{\beta}_{1j}^{\ O} = Y_{2j} - Y_{1j}$ (within-dyad difference)

$\hat{\gamma}_{00}$ = grand mean across all dyads

$\hat{\gamma}_{10}$ = average difference across all dyads

$\lambda_0 = \tau_{00}/(\tau_{00} + \sigma^2/2)$

$\lambda_1 = \tau_{11}/(\tau_{11} + 2\sigma^2)$

When τ_{00} (or τ_{11}) is large relative to σ^2: λ approaches 1, little shrinkage.

When τ_{00} (or τ_{11}) is small relative to σ^2: λ approaches 0, strong shrinkage toward grand mean.
