# Supplementary material for: Comparison of Two Analytical Approaches to Dyadic Illness Management Among Patient–Caregiver Dyads in Type 2 Diabetes
Source: Nurs Res. 2026 Feb 6;75(3):206–13. doi: 10.1097/NNR.0000000000000891 (PMC13098665; doi:10.1097/NNR.0000000000000891)

SUPPLEMENTAL DIGITAL CONTENT 2

Title

Comparison of Two Analytical Approaches to Dyadic Illness Management among Patient–Caregiver Dyads in Type 2 Diabetes

Supplemental Figure 1

Scatterplots of the correlation between predicted and observed dyadic average and incongruence across self-care/caregiver contribution to self-care behaviors (n = 251)

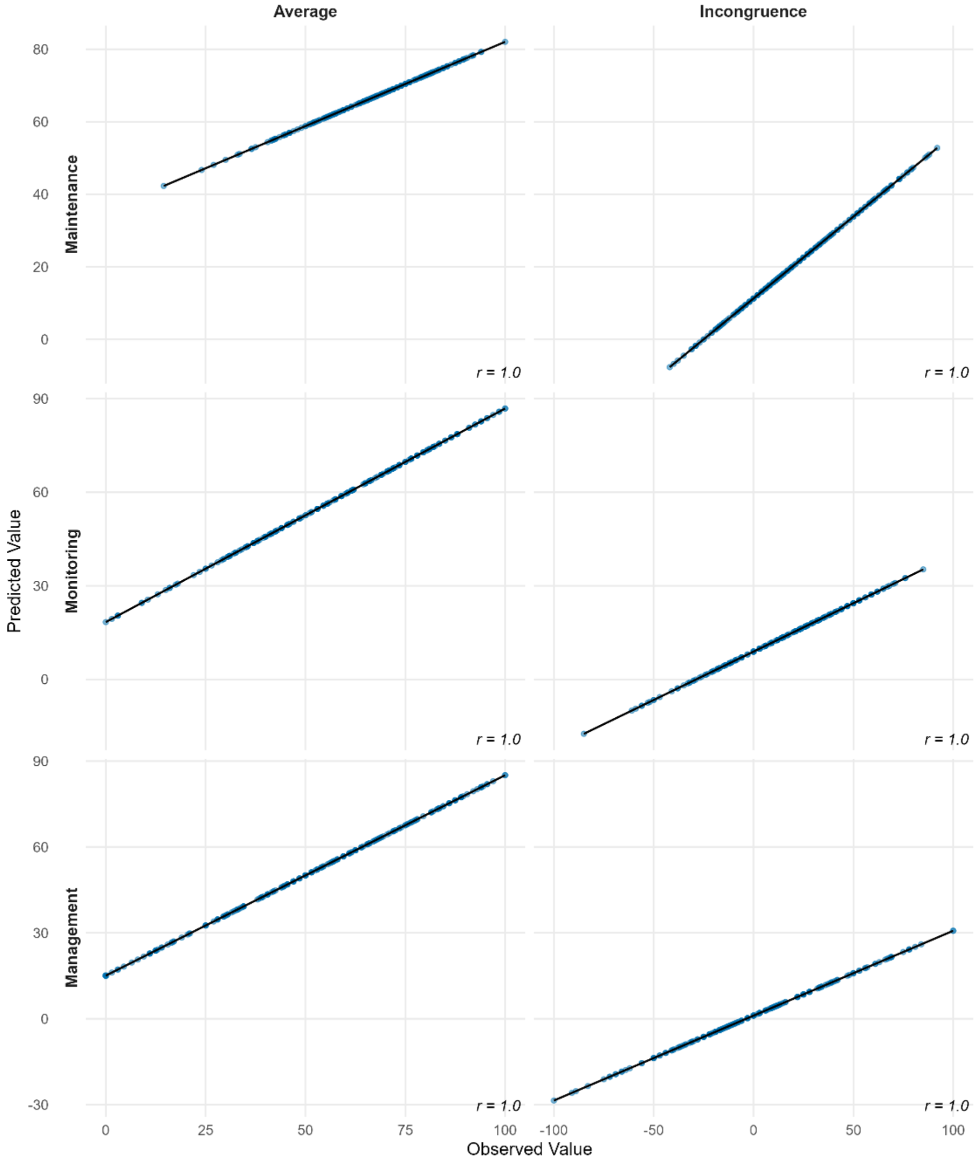

Supplement: Supplementary file 2 [file nnr-75-206-s002.pdf]
